# Supplementary material for: The mechanism of cell-cycle-dependent proteasomal degradation of archaeal ESCRT-III homolog CdvB in Sulfolobus
Source: EMBO J. 2026 Jan 9;45(4):1214–28. doi: 10.1038/s44318-025-00688-7 (PMC12909875; doi:10.1038/s44318-025-00688-7)
Supplement: Supplementary file 1 — Appendix [file 44318_2025_688_MOESM1_ESM.pdf]

Appendix for "The mechanism of cell cycle dependent proteasome-mediated CdvB degradation in *Sulfolobus*"

Yin-Wei Kuo[1,2], Jovan Traparić[1,2], Sherman Foo[1], Buzz Baum[1,3]

[1]Medical Research Council Laboratory of Molecular Biology; Cambridge CB2 0QH, United Kingdom

[2]These authors contributed equally to this work.

[3]Corresponding author. Email: bbaum@mrc-lmb.cam.ac.uk

Table of Contents

APPENDIX FIGURE S1 2

APPENDIX FIGURE S2 3

APPENDIX FIGURE S3 4

APPENDIX FIGURE S4 4

APPENDIX FIGURE S5 5

APPENDIX FIGURE S6 6

APPENDIX FIGURE S7 6

APPENDIX FIGURE S8 7

APPENDIX TABLE S1 7

APPENDIX TABLE S2 8

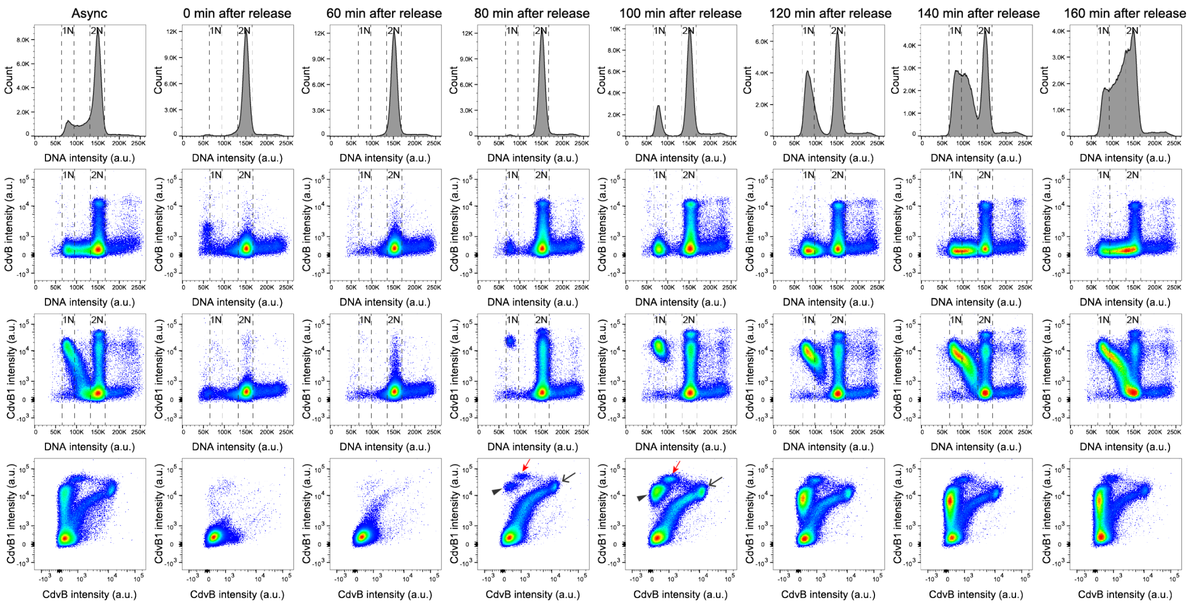

Appendix Figure S1. Example time-lapse flow cytometry histograms and scatter plots of synchronised MW001 control cells after release from acetic acid-induced G2 arrest. The scatter plots staining with CdvB (2nd row) and CdvB1 (3rd row) show that the degradation of CdvB occurs prior to the G1 phase entry (80 min and 100 min after release), while CdvB1 degradation predominantly occurs in G1/S phase (100 min to 160 min after release). The CdvB vs CdvB1 intensity scatter plots (bottom) show the time-course of CdvB degradation from the pre-constriction phase (high CdvB, high CdvB1 intensity; black arrows) through the constriction phase (low CdvB, high CdvB1 intensity; red arrows), into G1 (low CdvB, ~50% CdvB1 intensity of constriction phase, with 1N DNA content; arrow heads). The degradation timing observed is consistent with that inferred from the analysis of populations of asynchronous cells (left, and Fig. 1).

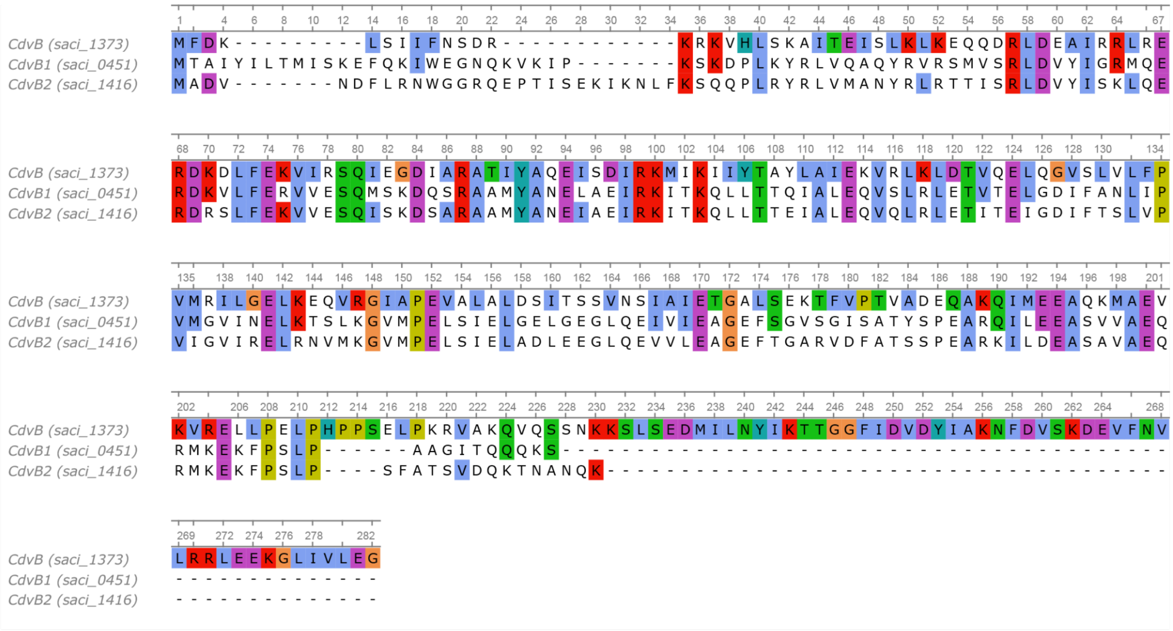

Appendix Figure S2. Sequence alignment of the ESCRT-III paralogs involved in *S. acidocaldarius* cell division. Sequence alignment by Clustal W of *Sulfolobus* CdvB homologs shows that the C-terminal ~50 aa of CdvB are absent in the other two *Sulfolobus* CdvB homologs. Sequences were obtained from Uniprot (CdvB: Q4J924; CdvB1: Q4JBG6; CdvB2: Q4J8Y4). The alignment figure was generated by Unipro UGENE.

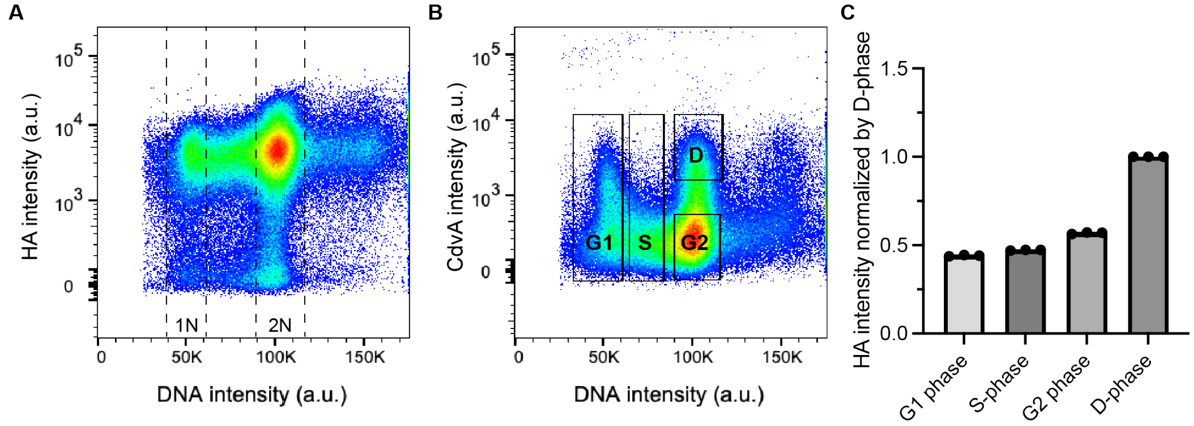

Appendix Figure S3. HA-LacS is not subject to cell cycle-dependent degradation at D/G1-phase transition. (A) Flow cytometry scatter plot of HA-LacS (4 hr after induction with 0.2% arabinose). **(B)** Image shows example of flow cytometry gates used to identify different cell cycle phases; n=2.5x10^5 events in each experiment. **(C)** Average HA signal intensity following HA-LacS overexpression for each cell cycle phase, normalized by levels in D-phase. This reveals that there is little cell cycle-dependent degradation of HA-LacS during the D/G1-phase transition.

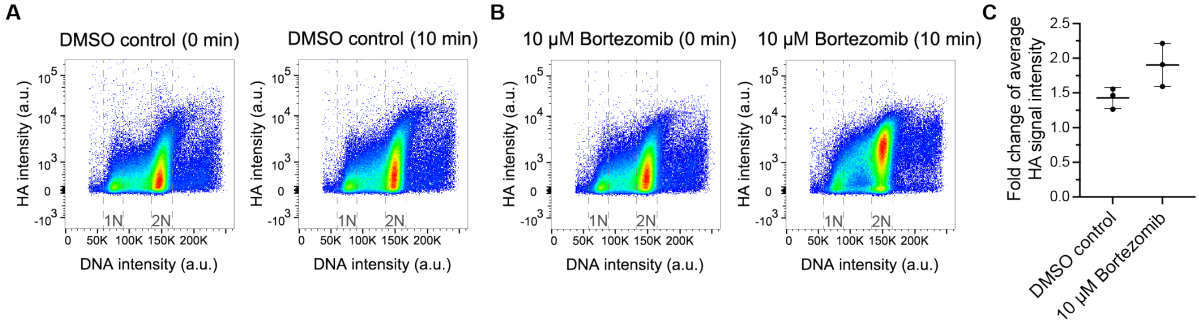

Appendix Figure S4. The C-terminal domain of CdvB is subject to degradation by the proteasome. (A, B) Example flow cytometry scatter plots of the HA-LacS-CdvB$^{C\text{-}term}$ overexpression strain (3.5 hr after arabinose induction) treated with DMSO (A) or 10 μM bortezomib (B). The HA signal reveals a rapid accumulation of HA-LacS-CdvB$^{C\text{-}term}$ within 10 min of proteasomal inhibition (n=2.5x10^5 events each). **(C)** Normalized HA signal intensity changes in cells with a 2N DNA content after 10 min of DMSO or bortezomib treatment, from 3 biological replicates (Student's t-test, p=0.07).

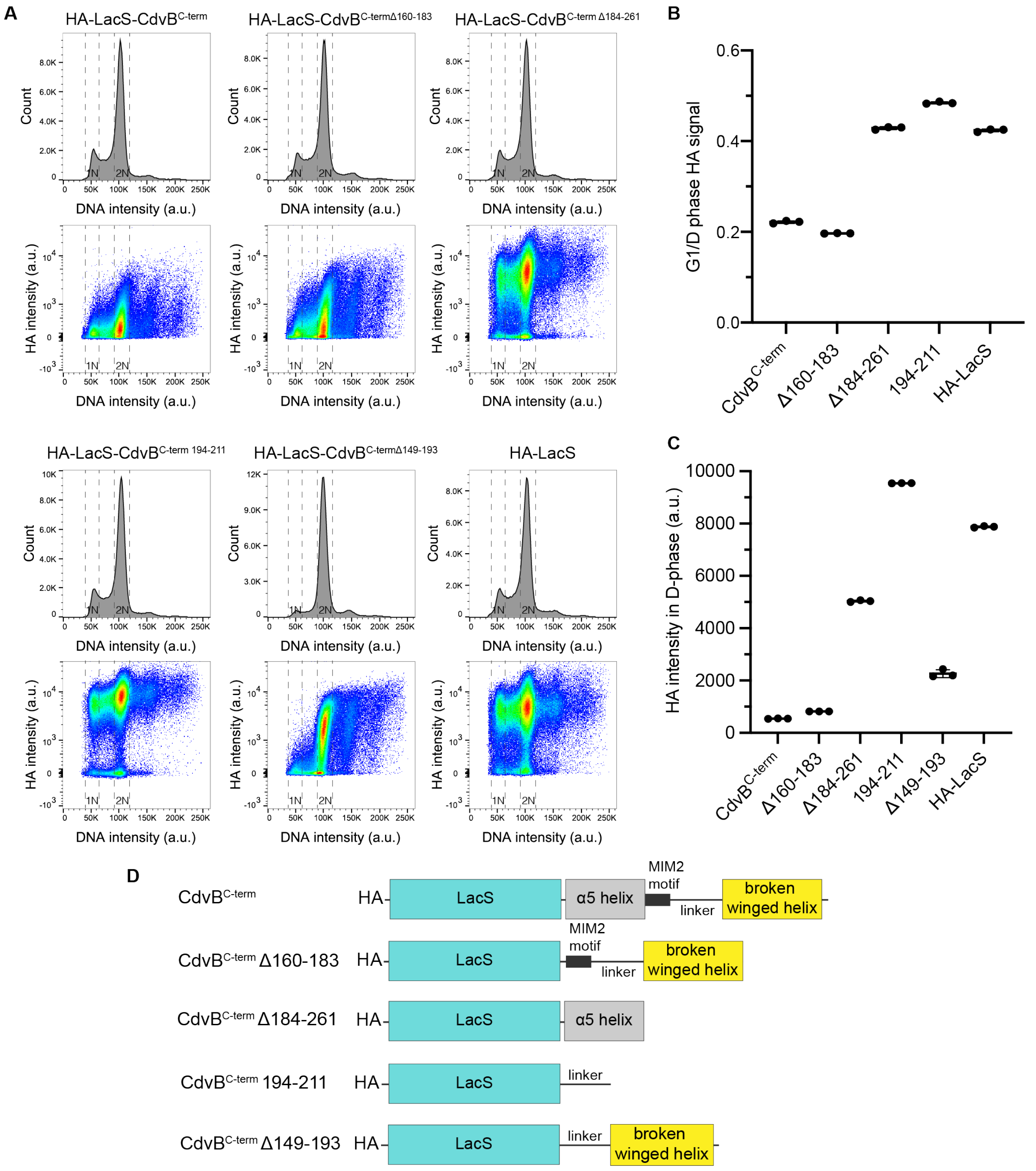

Appendix Figure S5. Roles of regions of the CdvB C-terminus in cyclic protein degradation. (A) Representative flow cytometry histograms and scatter plots for cells expressing HA-LacS fused to the different CdvB$^{C\text{-}term}$ truncation domains (n=2.5x10^5 events each). Note that the Δ149-193 construct shows a clear reduction in the percentage of cells with a 1N DNA content, indicating an arrest mid-division. **(B)** Ratio of HA intensity in G1/D-phase cells calculated from flow cytometry plots shown in (A). Note that the G1/D-phase HA ratio of Δ149-193 was not quantified due to the reduction in G1 population resulted from division defects. **(C)** Average HA signal intensity in D-phase measured from the same data. (D) Schematic diagram of the LacS-CdvB$^{C\text{-}term}$ constructs used in (A).

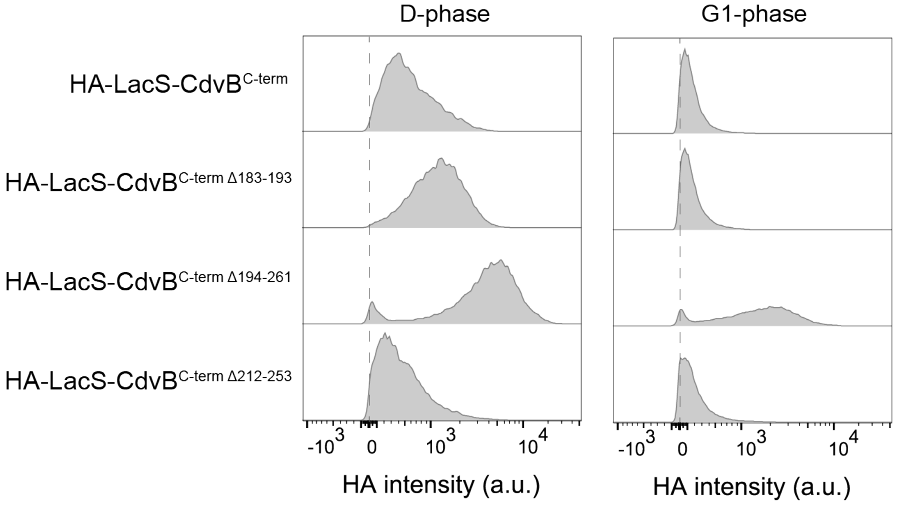

Appendix Figure S6. Representative histograms showing HA signal intensity for different CdvB tail truncations extracted from flow cytometry plots. Example histograms showing HA signal intensity in D-phase (left) and G1-phase (right) for each construct from Figure 3.

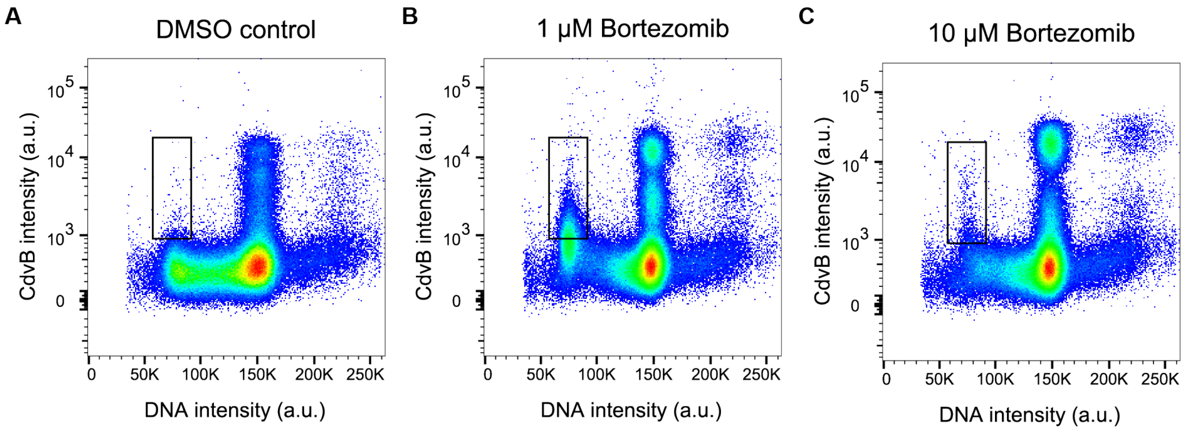

Appendix Figure S7. Partial inhibition of the proteasome prevents complete degradation of CdvB during passage through cytokinesis into G1. (A-C) Flow cytometry scatter plots of DMSO-treated control (A), 1 hr after low (1 μM) and high concentration (10 μM) of bortezomib-treated cells in (B) and (C) respectively. The rectangular box highlights the CdvB positive cells in G1 phase (n=2.5x10^5 events each). Note that a high dosage (10 μM) of Bortezomib leads to a strong inhibition of CdvB degradation and arrests the cells at pre-constriction phase during division (Tarrason Risa *et al.* 2020). This strong proteasomal inhibition prevents the entry of G1 and reduces the percentage of G1 cells with residual CdvB, which are therefore more evident in (B).

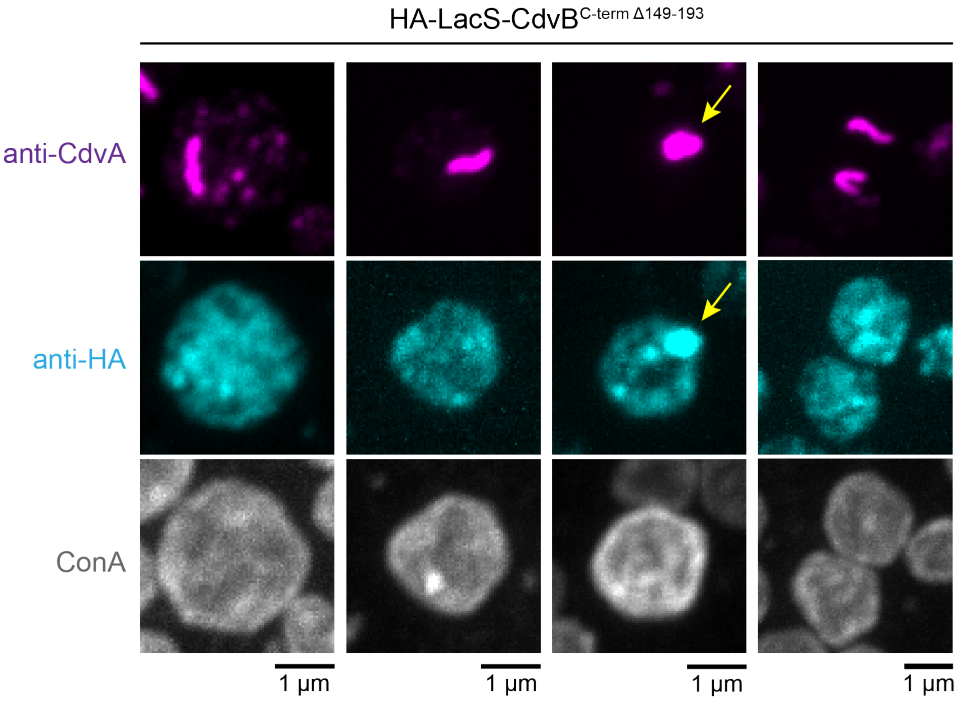

Appendix Figure S8. Localization of CdvA and HA in cells expressing HA-LacS-CdvB$^{C\text{-term}\Delta149\text{-}193}$. Example immunofluorescence images of cells expressing HA-LacS-CdvB$^{C\text{-term}\Delta149\text{-}193}$ from arabinose-inducible promoter (4 hr induction) imaged by spinning disk confocal microscopy. Cell surface was stained by fluorophore-conjugated Concanavalin A (ConA). The HA signal appears diffuse in most cells with few cells showing partial colocalization with foci of CdvA (yellow arrows). Note that complete CdvA ring was rarely observed in this condition.

Appendix Table S1: Antibodies used in this study

**Antibody**	**Host organism**	**Dilution**	**Catalogue number**
Anti-CdvB serum	Rabbit	1:1000	-
Anti-CdvB1 IgY	Chicken	1:1000	-
Anti-CdvB2 IgG (peptide antibody)	Guinea Pig	1:1000	-
Anti-CdvA IgY	Chicken	1:1000	-
Anti-CdvA serum	Rabbit	1:1000	-
Anti-PAN serum	Rabbit	1:2000	-
Anti-HA Monoclonal Antibody (2-2.2.14)	Mouse	1:1000-1:5000	26183 (Invitrogen)
Anti-Alba serum	Rabbit	1:2000	-
Anti-Rabbit IgG, AF488	Goat	1:1000	A11034 (Invitrogen)
Anti-Mouse IgG, AF488	Goat	1:200-1:1000	A11029 (Invitrogen)

Anti-Mouse IgG, AF647	Goat	1:200-1:1000	A21235 (Invitrogen)
Anti-Chicken IgY, AF546	Goat	1:200-1:1000	A11040 (Invitrogen)
Anti-Chicken IgY, AF647	Goat	1:1000	A21449 (Invitrogen)
Anti-Guinea Pig IgG, AF546	Goat	1:200-1:1000	A11074 (Invitrogen)
Anti-Mouse IgG IRDye 800CW	Goat	1:10,000	926-32210 (LI-COR Biosciences)
Anti-Rabbit IgG IRDye 800CW	Goat	1:10,000	926-32211 (LI-COR Biosciences)

Appendix Table S2: Primer sets used for RT-qPCR

**Target gene**	**Forward primer (5’ to 3’)**	**Reverse primer (5’ to 3’)**
SecY (saci_0574)	ACTCTTGCTTGACGAGATGATAC	ACTCTGTACGGAGACTATTCCA
CdvB (saci_1373)	ACTGGTGCATTAAGCGAGAA	TTGGTAACTCTGAAGGTGGATG
PAN (saci_0656)	CCCTGGCACTGGAAAAACCA	GCAGCAATCGCATCAAGCTCA
