# Supplementary figures and images for: The mechanism of cell-cycle-dependent proteasomal degradation of archaeal ESCRT-III homolog CdvB in Sulfolobus
Source: EMBO J. 2026 Jan 9;45(4):1214–28. doi: 10.1038/s44318-025-00688-7 (PMC12909875; doi:10.1038/s44318-025-00688-7)

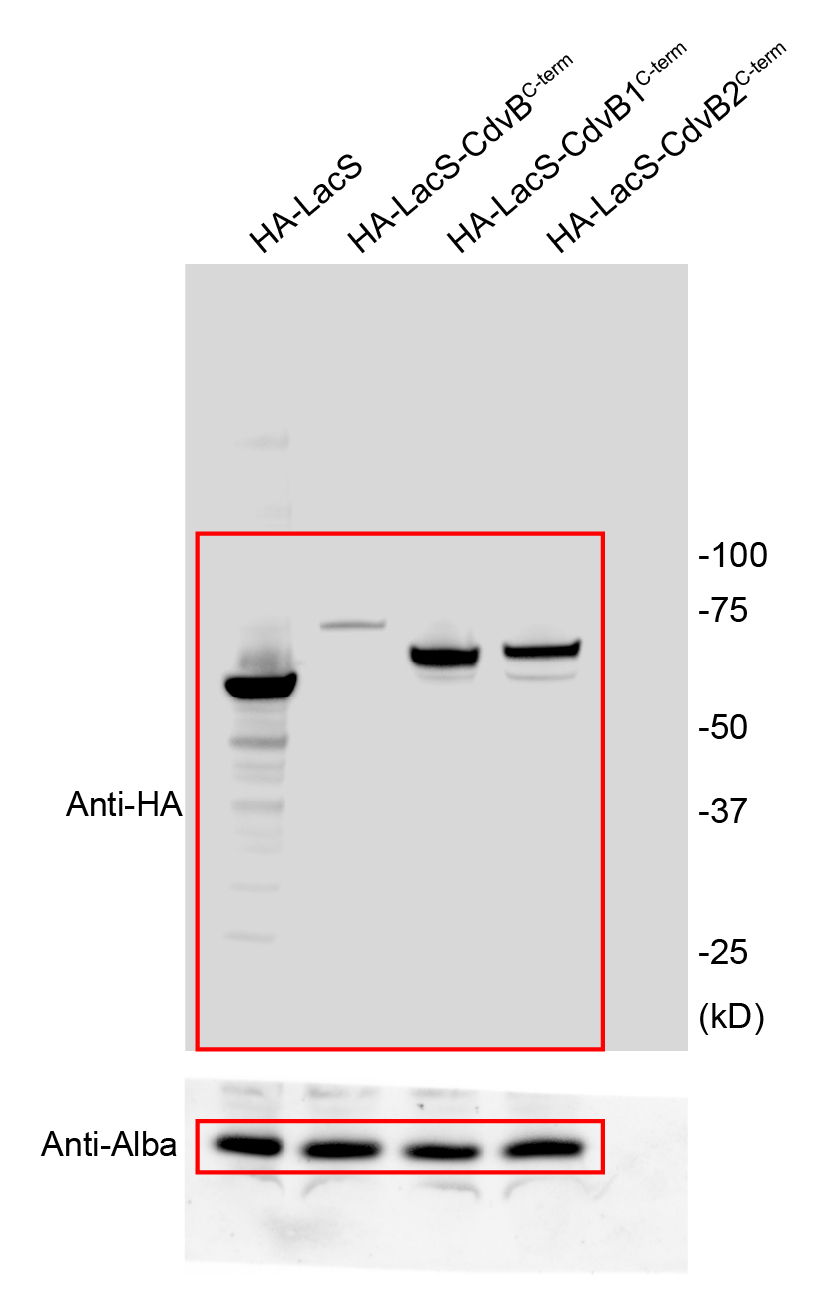

Supplement: Supplementary file 4 — Source data Fig. 2 [file 44318_2025_688_MOESM4_ESM.zip › Figure 2/2A/WesternBlot_anti-HA_anti-Alba.jpg]

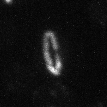

Supplement: Supplementary file 4 — Source data Fig. 2 [file 44318_2025_688_MOESM4_ESM.zip › Figure 2/2F/CdvB-HA_OE-3.tif]

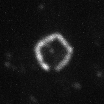

Supplement: Supplementary file 4 — Source data Fig. 2 [file 44318_2025_688_MOESM4_ESM.zip › Figure 2/2F/CdvB-HA_OE-2.tif]

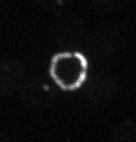

Supplement: Supplementary file 4 — Source data Fig. 2 [file 44318_2025_688_MOESM4_ESM.zip › Figure 2/2F/CdvB-HA_OE-1.tif]

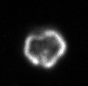

Supplement: Supplementary file 4 — Source data Fig. 2 [file 44318_2025_688_MOESM4_ESM.zip › Figure 2/2E/HA-LacS-CdvB-Cterm-1.tif]

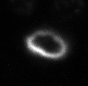

Supplement: Supplementary file 4 — Source data Fig. 2 [file 44318_2025_688_MOESM4_ESM.zip › Figure 2/2E/HA-LacS-CdvB-Cterm-3.tif]

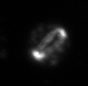

Supplement: Supplementary file 4 — Source data Fig. 2 [file 44318_2025_688_MOESM4_ESM.zip › Figure 2/2E/HA-LacS-CdvB-Cterm-2.tif]

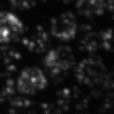

Supplement: Supplementary file 6 — Source data Fig. 4 [file 44318_2025_688_MOESM6_ESM.zip › Figure 4/4D/MW001_control_2.tif]

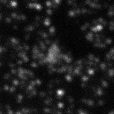

Supplement: Supplementary file 6 — Source data Fig. 4 [file 44318_2025_688_MOESM6_ESM.zip › Figure 4/4D/MW001_control_3.tif]

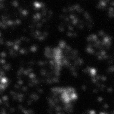

Supplement: Supplementary file 6 — Source data Fig. 4 [file 44318_2025_688_MOESM6_ESM.zip › Figure 4/4D/MW001_control_1.tif]

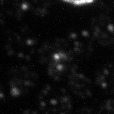

Supplement: Supplementary file 6 — Source data Fig. 4 [file 44318_2025_688_MOESM6_ESM.zip › Figure 4/4D/MW001_control_4.tif]

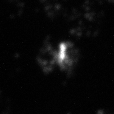

Supplement: Supplementary file 6 — Source data Fig. 4 [file 44318_2025_688_MOESM6_ESM.zip › Figure 4/4D/PANE237Q_OE_1.tif]

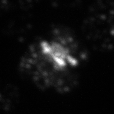

Supplement: Supplementary file 6 — Source data Fig. 4 [file 44318_2025_688_MOESM6_ESM.zip › Figure 4/4D/PANE237Q_OE_3.tif]

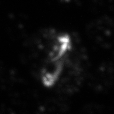

Supplement: Supplementary file 6 — Source data Fig. 4 [file 44318_2025_688_MOESM6_ESM.zip › Figure 4/4D/PANE287Q_OE_4.tif]

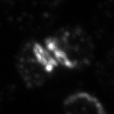

Supplement: Supplementary file 6 — Source data Fig. 4 [file 44318_2025_688_MOESM6_ESM.zip › Figure 4/4D/PANE287Q_OE_2.tif]

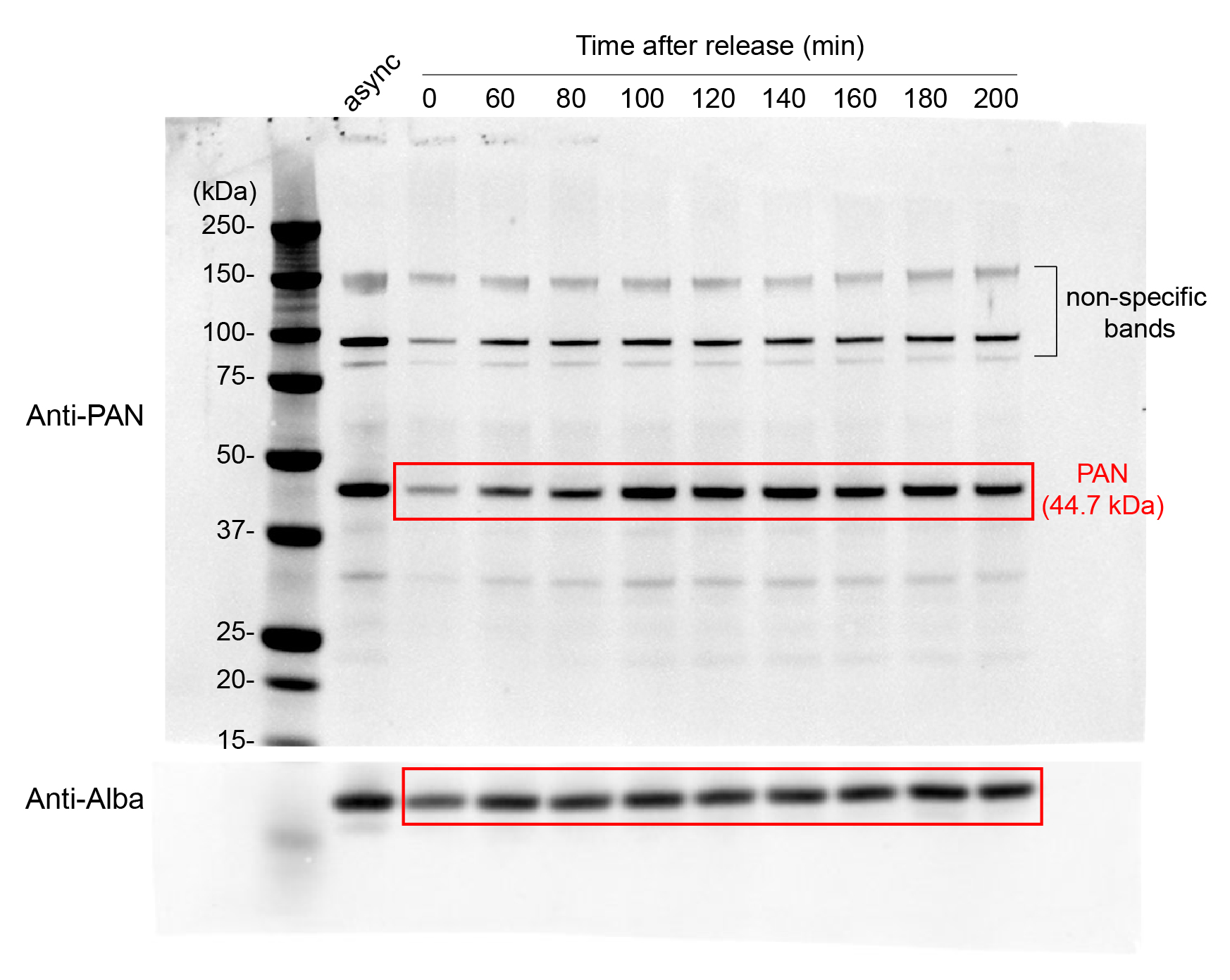

Supplement: Supplementary file 7 — Source data Fig. 5 [file 44318_2025_688_MOESM7_ESM.zip › Figure 5/5A/Synchronization_WB_anti-PAN_anti-Alba.jpg]

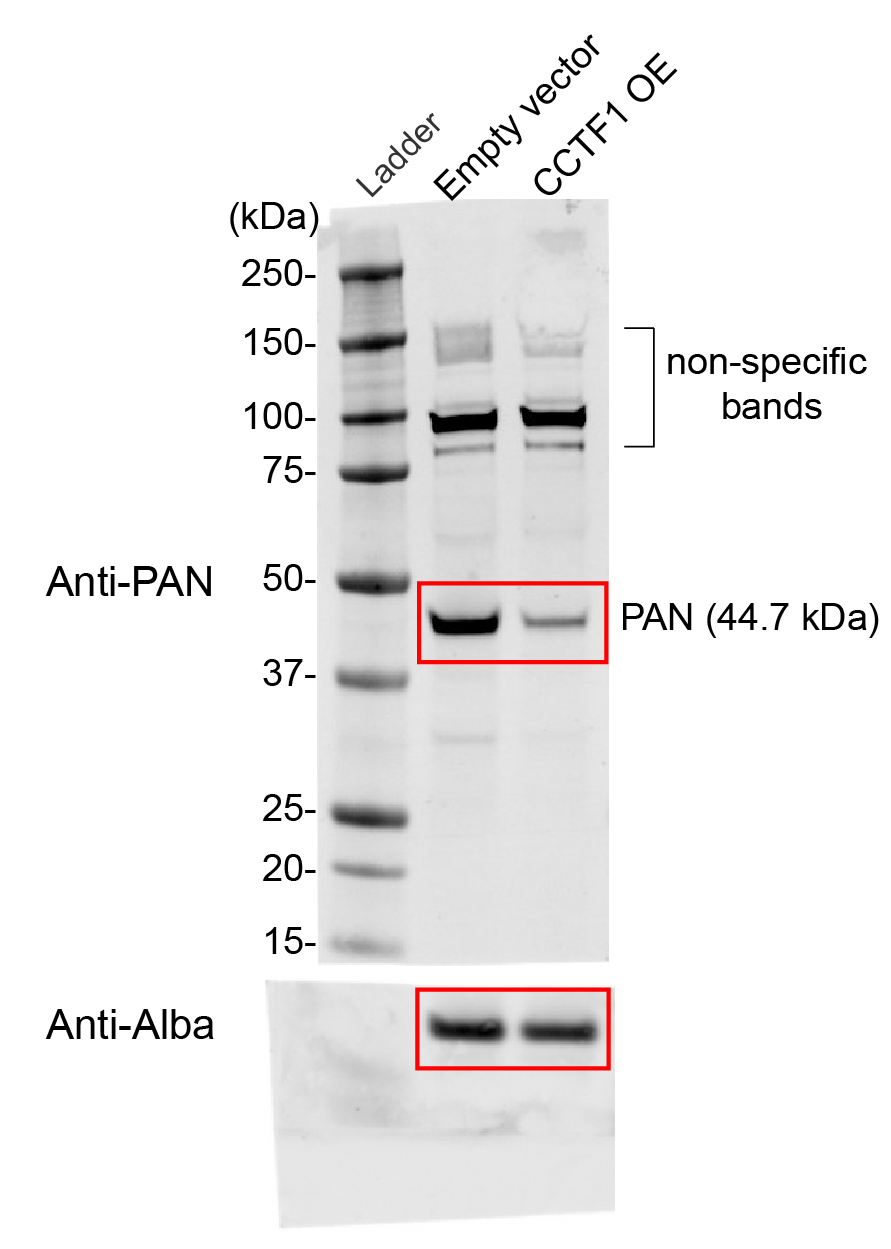

Supplement: Supplementary file 8 — Source data Fig. 6 [file 44318_2025_688_MOESM8_ESM.zip › Figure 6/6E/Fig6E_WesternBlot-01-01.jpg]

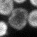

Supplement: Supplementary file 8 — Source data Fig. 6 [file 44318_2025_688_MOESM8_ESM.zip › Figure 6/6G_IF/CCTF1_OE-1.tif]

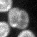

Supplement: Supplementary file 8 — Source data Fig. 6 [file 44318_2025_688_MOESM8_ESM.zip › Figure 6/6G_IF/Empty_vector-3.tif]

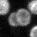

Supplement: Supplementary file 8 — Source data Fig. 6 [file 44318_2025_688_MOESM8_ESM.zip › Figure 6/6G_IF/CCTF1_OE-2.tif]

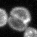

Supplement: Supplementary file 8 — Source data Fig. 6 [file 44318_2025_688_MOESM8_ESM.zip › Figure 6/6G_IF/Empty_vector-1.tif]

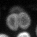

Supplement: Supplementary file 8 — Source data Fig. 6 [file 44318_2025_688_MOESM8_ESM.zip › Figure 6/6G_IF/CCTF1_OE-3.tif]

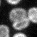

Supplement: Supplementary file 8 — Source data Fig. 6 [file 44318_2025_688_MOESM8_ESM.zip › Figure 6/6G_IF/Emptvy_vector-2.tif]
